# Trends regarding percutaneous endoscopic gastrostomy: A nationwide population-based study from 1997 to 2010

**DOI:** 10.1097/MD.0000000000003910

**Published:** 2016-06-17

**Authors:** Wei-Kuo Chang, Kuen-Tze Lin, Chen-Liang Tsai, Chi-Hsiang Chung, Wu-Chien Chien, Chun-Shu Lin

**Affiliations:** a Division of Gastroenterology, Department of Internal Medicine; b Department of Radiation Oncology; c Division of Pulmonary and Critical Care Medicine, Department of Internal Medicine, Tri-Service General Hospital, National Defense Medical Center; d School of Public Health, National Defense Medical Center; e Department of Medical Research, Tri-Service General Hospital, National Defense Medical Center, Taipei, Taiwan, ROC.

**Keywords:** enteral nutrition, medical service, mortality, percutaneous endoscopic gastrostomy

## Abstract

Percutaneous endoscopic gastrostomy (PEG) is widely used in patients requiring long-term tube feeding. Traditional PEG studies usually focused on practical, technical, and ethical issues. There have been little epidemiological studies on PEG utilization and services in Asia. We evaluated the changes in PEG utilization, patient selection, patient characteristics, and medical service in Taiwan from 1997 to 2010.

This retrospective study analyzed the data of patients admitted for PEG tube placement according to the International Classification of Diseases, Ninth Revision (procedure code 43.11) extracted from the National Health Insurance database between 1997 and 2010.

From 1997 to 2010, the incidence of PEG increased from 0.1 to 3.8/10^5^ population and incidence of PEG among aged patients increased from 0.9 to 19.0/10^5^ population. Compared 1997–2004 to 2005–2010 periods, the percentage of cerebrovascular diseases decreased and esophageal cancer increased in the later period. PEG was mainly performed in male patients and at medical centers. Medical costs, Charlson Comorbidity Index (CCI) scores, and post-PEG mortality rates were higher in the 2005–2010 period than in the 1997–2004 period.

PEG procedures are being increasingly performed in Taiwan, and changes in patient selection were noted. The seriousness of accompanying diseases, medical costs, and post-PEG mortality rates in patients undergoing PEG has increased. The present findings may help in the implementation of PEG, relocation of medical resources, and improvement of PEG-related care.

## 1 Introduction

Percutaneous endoscopic gastrostomy (PEG) was introduced in 1980,^[1]^ and has since been widely used in patients requiring long-term enteral nutrition. The number of PEG procedures increased from 61,000 in 1989 to 216,000 in 2000, making PEG the second most common indication for endoscopy of the upper gastrointestinal tract.^[2,3]^ Over 17,000 PEG procedures are performed annually in the UK.^[4]^

However, traditional PEG studies usually focused on practical, technical, and ethical issues.^[5,6]^ There have been little epidemiological studies on PEG utilization and services in Asia.^[4,7,8]^

The Taiwan National Health Insurance (NHI) program has been operating since 1995 and covers approximately 99% of the entire population.^[9]^ The large sample size of the hospital discharge database will enable us to perform a descriptive epidemiologic study, and develop a strategy for improvement of PEG-related medical service. The aim of this study was to evaluate the changes in PEG utilization, patient selection, patient characteristics, and medical service in Taiwan from 1997 to 2010.

## 2 Methods

### 2.1 Research database

This retrospective study analyzed data from the NHI database. The NHI program is reinforced by related laws, and all facilities offering medical services are obligated to claim medical fees every month from the NHI administration. These claim records are entered into the NHI research database, and this database has been proven to be one of the most representative and detailed databases for studies.^[10]^ We obtained access to the NHI research database, and our study protocol was approved by the research ethics committee of the institute.

### 2.2 Study population

All hospitalization records from the NHI research database between January 1, 1997 and December 31, 2010 were analyzed (N = 2,029,528), and data of patients admitted for PEG tube placement according to the International Classification of Diseases, Ninth Revision (procedure code 43.11) were included in the study.

### 2.3 Study variables

Data on age, sex, indications for PEG, hospital level, geographical region, extent of urbanization, medical cost, length of hospital stay, the Charlson Comorbidity Index (CCI), and post-PEG mortality rate were extracted from the NHI research database. The CCI indicated serious accompanying diseases. To calculate the CCI, the first 5 diagnostic codes (N-Code) of a patient were each multiplied by the scores assigned to 19 different diseases mentioned by Charlson et al.^[11]^ The mortality rate was assessed at 3, 7, 14, 30, 45, 60, 180, and 360 days after PEG.

### 2.4 Statistical analysis

Parametric continuous data were compared using the Student *t* test, and categorical data were compared using the Chi-square test and Yates correction or Fisher exact test. Linear regression was used to analyze trends from 1997 to 2010. Statistical analyses were performed using SPSS 22.0 software. A *P*-value <0.05 was considered statistically significant.

## 3 Results

### 3.1 Patient characteristics

**Figure 1:**
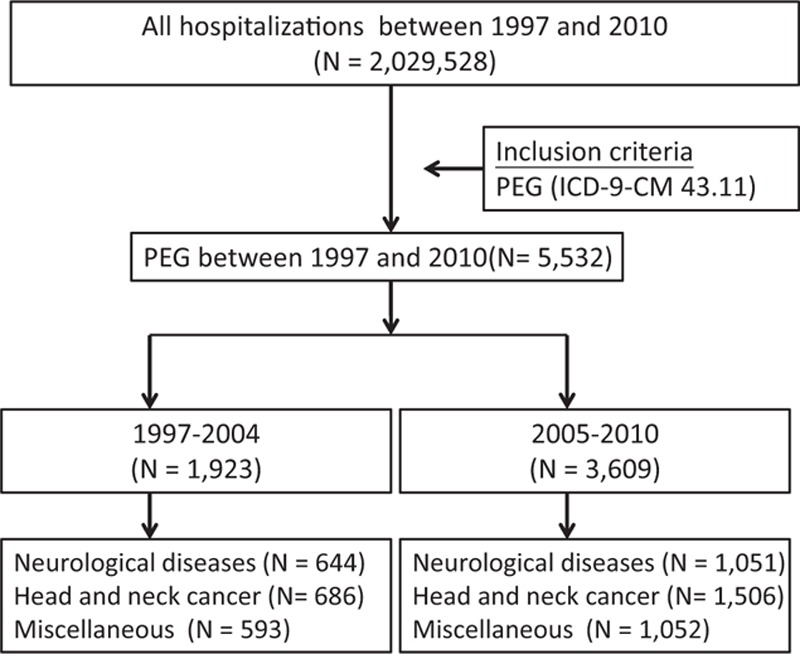
Flow chart diagram for the selection of the study sample from the National Health Insurance Research Database in Taiwan. PEG = percutaneous endoscopic gastrostomy.

The patients who underwent PEG were arbitrary divided into those who underwent the procedure in 1997 to 2004 (n = 1923) and those who underwent the procedure in 2005 to 2010 (n = 3609) (Fig. 1; Table 1).^[12]^ From 1997 to 2010, the annual number of patients who underwent PEG increased from 25 to 886 and incidence of PEG increased from 0.1 to 3.8/10^5^ population (Table 2). The distribution of sex and age was similar between the 1997 to 2004 and 2005 to 2010 periods (Table 3).

**Table 1:**
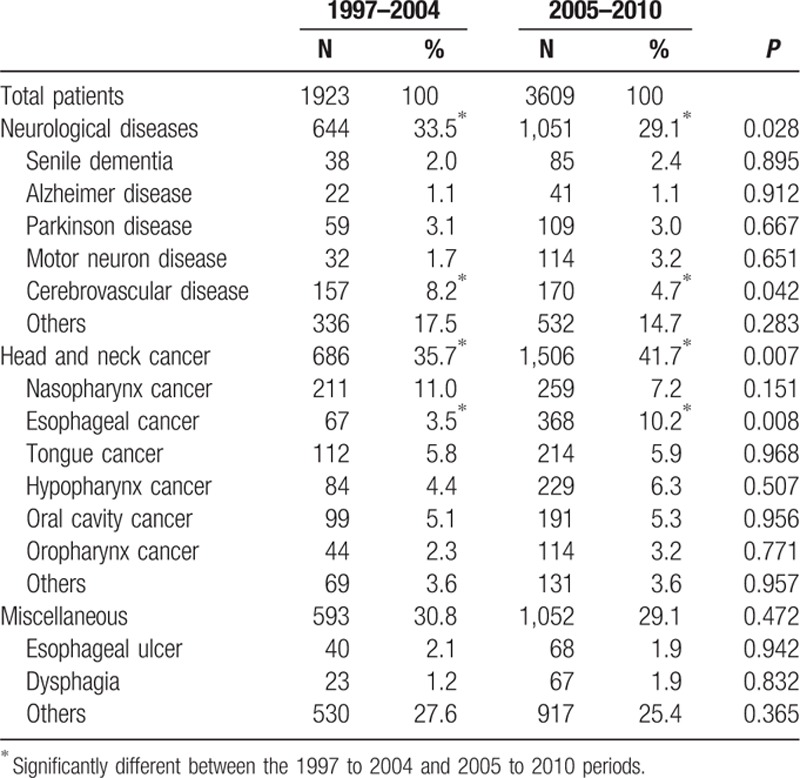
Indications for percutaneous endoscopic gastrostomy.

**Table 2:**
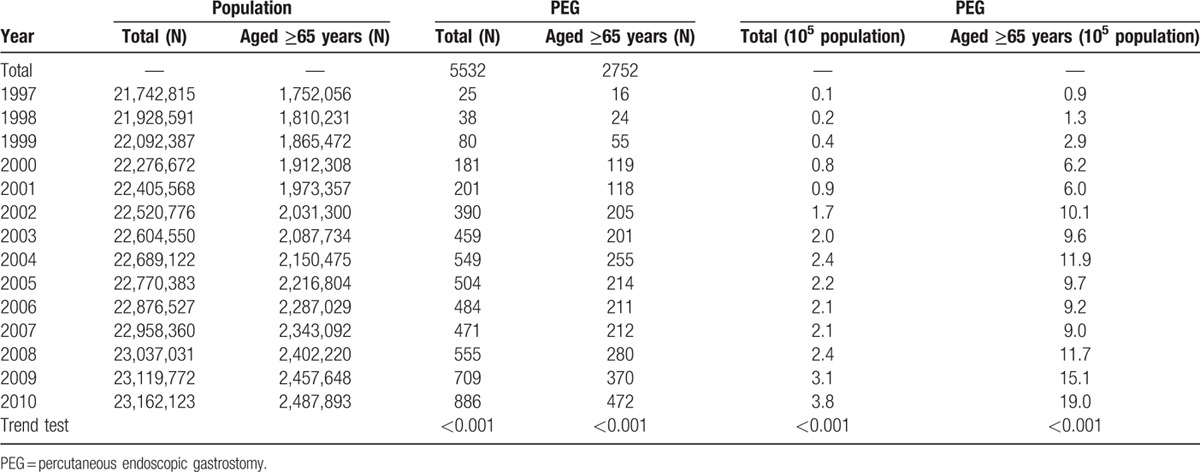
Trends in the number of PEG procedures.

**Table 3:**
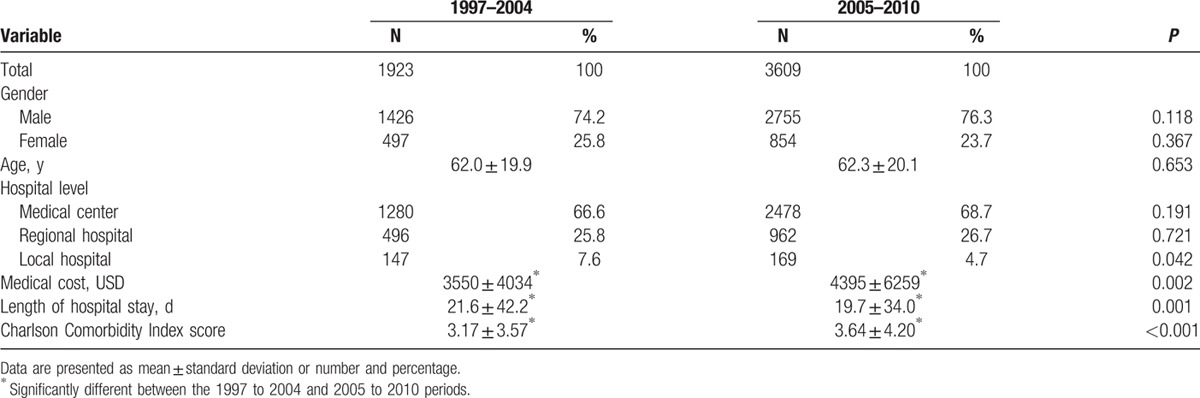
Patient characteristics.

### 3.2 Patient selection

Compared between 1997 to 2004 and 2005 to 2010 periods, patients who underwent PEG in the later period has significantly lower percentages of neurological diseases (29.1% vs 33.5%, *P* = 0.028) and cerebrovascular diseases (4.7% vs 8.2%, *P* = 0.042); but has higher percentage of head and neck cancer (41.7% vs 35.7%, *P* = 0.007) and esophageal cancer (10.2% vs 3.5%, *P* = 0.008).

**Figure 2:**
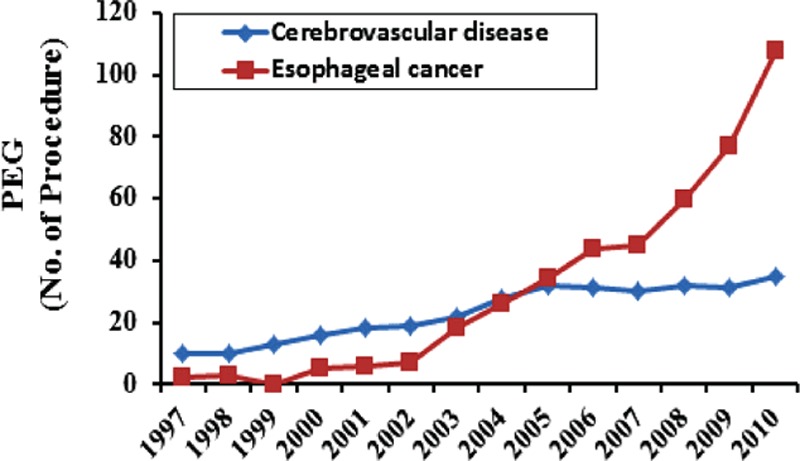
Trends in percutaneous endoscopic gastrostomy performed in patients with cerebrovascular diseases (trend test, *P* < 0.001) and esophageal cancer (trend test, *P* < 0.001) in the 1997 to 2010 period. PEG = percutaneous endoscopic gastrostomy.

During the study period, the annual number of patients with cerebrovascular diseases who underwent PEG gradually increased from 10 to 35; however, the percentage of these patients who underwent PEG decreased from 40% to 4.0% (Fig. 2). The annual number of patients with esophageal cancer who underwent PEG rapidly increased from 2 to 108, and the percentage of these patients who underwent PEG increased from 8.0% to 12.2% (Fig. 2).

**Figure 3:**
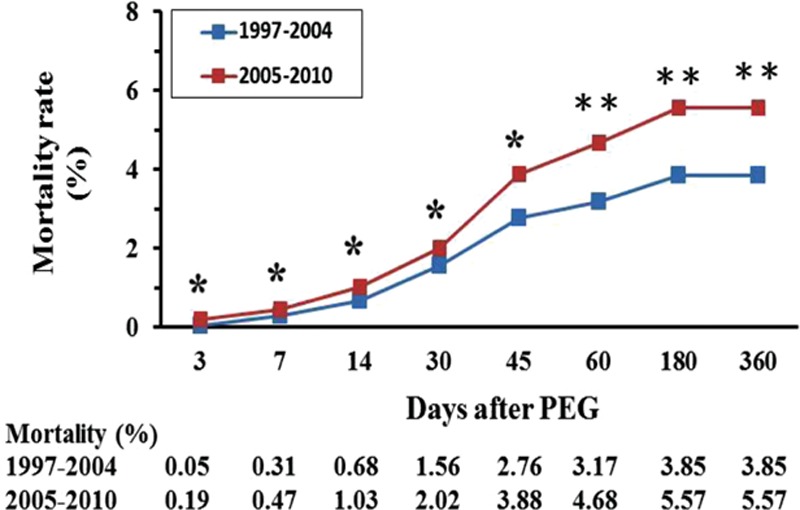
Mortality rates measured at 3, 7, 14, 30, 45, 60, 180, and 360 days after percutaneous endoscopic gastrostomy (^∗^*P* < 0.05, ^∗∗^*P* < 0.001). PEG = percutaneous endoscopic gastrostomy.

Compared between 1997 to 2004 and 2005 to 2010 periods, patients who underwent PEG in the later period has significantly lower percentages in local hospitals (4.7% vs 7.6%, *P* = 0.042) and significantly lower length of hospital stay (19.7 ± 34.0 vs 21.6 ± 42.2, *P* = 0.001); but has significantly higher medical costs (4395 ± 6259 vs 3550 ± 4034, *P* = 0.002) and higher CCI (3.17 ± 3.57 vs 3.64 ± 4.20, *P* < 0.001). The mortality rates at 3, 7, 14, 30, 45, 60, 180, and 360 days after PEG were all significantly higher in the 2005 to 2010 period (Fig. 3).

**Figure 4:**
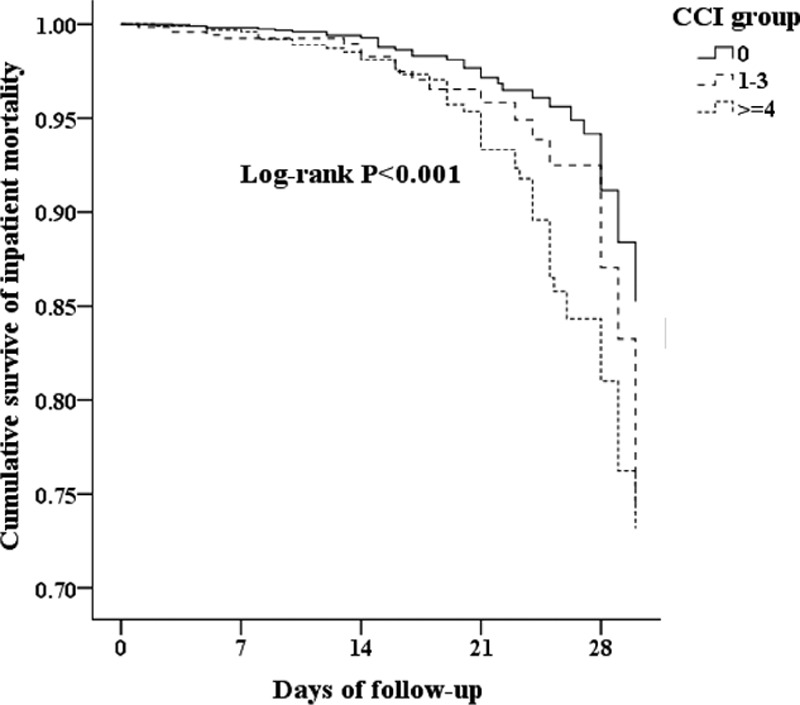
Survival rates between different CCI groups. CCI = Charlson Comorbidity Index (*P* < 0.001).

We analyzed the mortality rates with variable factors between these 2 periods (Table 4). We noticed that patients in local hospital (*P* = 0.042), high- and middle-level urbanization (*P* < 0.001), and with high CCI (*P* < 0.001, Fig. 4) sustained worse survival.

**Table 4:**
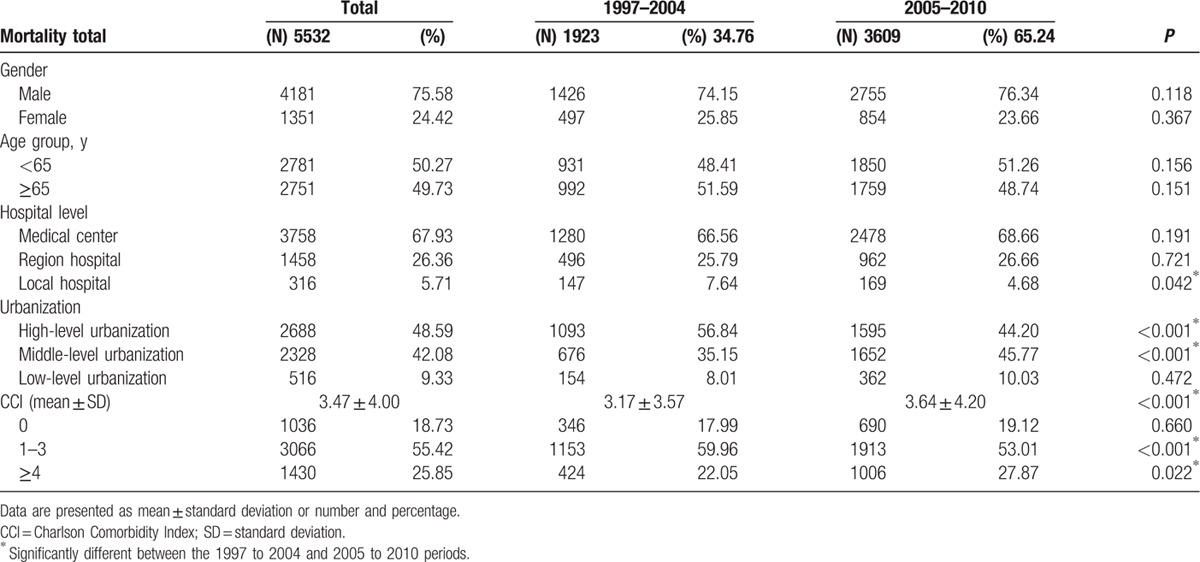
Mortality rates.

## 4 Discussion

The present study found several potentially significant variations in PEG implantation, which may help identify and overcome problems in the utilization of PEG. A continuous increase in the number of PEG procedures was noted over the study period. The use of PEG is influenced by sex and the presence of a nearby medical center. Changes in the trend of common indications, such as cerebrovascular disease and head/neck cancer, for PEG were noted. More serious accompanying diseases, and higher medical costs and post-PEG mortality rates were present among the patients who underwent PEG in the 2005 to 2010 period than in the 1997 to 2004 period.

### 4.1 Increasing number of PEG procedures

In 1997, Taiwan had a population of 21,742,815, and 8.1% of the population was aged over 65 years. The proportion of the population aged over 65 years is estimated to dramatically increase from 11% in 2010 to 17% in 2017 and 20% in 2025. The overall life expectancy in Taiwan was 80.06 years in 2012, and it ranked 30th in the world for life expectancy.^[13]^ The increasing number of PEG procedures being performed reflects the increasing demand for PEG to treat patients with neurological diseases and head and neck malignancies.

### 4.2 Changes in patient selection

During the 1997 to 2004 period, the leading diseases for PEG were nasopharynx cancer (11.0%), cerebrovascular diseases (8.2%), tongue cancer (5.8%), and oral cavity cancer (5.1%). We noted changes in the trend of common indications, such as cerebrovascular disease and head/neck cancer, for PEG over the 14-year study period.

During the study period, the annual number of patients with cerebrovascular diseases who underwent PEG gradually increased; however, the percentage of these patients among those who underwent PEG dramatically decreased. This finding is consistent with the findings of previous study in Taiwan, which showed a low prevalence of PEG of 0.1% in nursing home residents in 2002 and 2.8% in 2007.^[6,14]^ However, high prevalences of PEG of 6.6% in nursing home residents in Germany, 33.3% in Israel, and 0% to 38.9% in the US have been reported.^[15–18]^

Yeh et al^[19]^ demonstrated that traditional family members or surrogate decision makers strongly followed Chinese culture values. Many elderly patients have neurological diseases such as dementia or cognitive impairment. However, surrogate decision makers, who are likely to maintain body integrity and end-of-life stability, do not accept PEG tube placement for long-term tube feeding, reflecting the slow increase in the number of PEG procedures performed in patients with neurological and cerebrovascular diseases from 1997 to 2010.

Head and neck cancer patients represent a unique population that can benefit from PEG. PEG can be performed not only as a means of receiving nutrition, but also as palliative care for malignant obstructions of the gastrointestinal tract.^[20,21]^ Most of these patients are conscious, and capable of receiving information and providing consent. They are usually requested to participate in the decision-making for treatment choices. In these patients, PEG is associated with comfort for the patient, easy maintenance of adequate nutrition, and good tolerance of a complete chemo radiotherapy regimen.^[22]^ The present study clearly demonstrated a continuous increase in the number of PEG procedures performed in patients with head and neck cancer over the 14-year study period.

### 4.3 Nonevidence-based indications

The PEG procedure is not technically difficult. Many physicians have a low threshold for PEG tube placement. The demand for PEG has increased and has extended to areas where the benefits of PEG were previously uncertain. PEG has been used for patients with aspiration pneumonia, esophageal ulcer, and bowel obstruction. A high proportion of patients with “other” conditions reflects the controversial issue of nonevidence-based indications. Janes et al^[23]^ showed that nonevidence-based indications for PEG increased from 16% in 1992 to 31% in 2002, which was associated with a significant increase in the 30-day mortality. This finding shows the need for appropriate guidelines on PEG and education courses on PEG for physicians, which will help in patient selection. Recently, a review article from Spain explored a practical overview on the indications of PEG.^[24]^ The indications include neurological diseases, and head and neck cancer which are the same as ours. However, our indication did not cover few situations that were mentioned in this review article, for example, chemotherapy in oncologic disease, scleroderma, cystic fibrosis, etc.

### 4.4 Disparities in access to PEG

Disparities in access to PEG, according to sex, race, geographical variation, and extent of urbanization have been previously reported.^[16,18,25–28]^ The present study demonstrated that PEG was mainly performed in male patients (74.2%–76.3%) and at medical centers (66.6%–68.7%). This indicates that the use of PEG is influenced by sex, the presence of a nearby medical center rather than by objective clinical assessments.

### 4.5 Quality of PEG

In Taiwan, PEG has been reported to have positive outcomes related to good nutritional status, low complication rates, low procedure-related mortality (0%–0.09%), relatively low 30-day mortality (1.86%–3.3%), and relatively high patient satisfaction (70%).^[6,14,19,29]^

Patients undergoing PEG may have comorbid illnesses, may not be able to tolerate hemodynamic disturbances, and may amplify the challenges of patient care. In the present study, we found that patients who underwent PEG had more serious accompanying diseases, higher medical cost, and higher post-PEG mortality rates in the 2005 to 2010 period than in the 1997 to 2004 period. PEG is generally considered as a simple and safe minimally invasive procedure, and endoscopists can not only act as technicians, but also evaluate the underlying diseases and clinical outcomes.^[4]^ The trend of increasing post-PEG mortality rates raises concerns on whether the selection of some patients is inappropriate and whether PEG can achieve long-term enteral nutritional goals while maintaining a low postprocedure mortality rate.

### 4.6 Limitations

The present study has some limitations. This retrospective study could not accurately collect clinical information, such as albumin levels, nutritional status, prophylactic antibiotic use, and procedure-related complications, which is an inherent limitation in all retrospective studies. The indications were estimated and may not accurately reflect the actual reasons for PEG. Because of single country analysis, the results from this research may not be generalized to other countries and cultural environments.

## 5 Conclusions

PEG procedures are being increasingly performed in Taiwan. Changes in the trend of common indications, such as cerebrovascular disease and head/neck cancer, for PEG are present. Although affordable and high quality PEG services are available, access to PEG is not equal in Taiwan. Additionally, the seriousness of accompanying diseases, medical costs, and post-PEG mortality rates in patients undergoing PEG has increased recently. Financial benefits should be provides, practice guidelines on PEG should be introduced, and appropriate communication among patients, caregivers, and healthcare personnel should be encouraged to improve the quality of PEG and increase its use. The findings of the present study may help in the implementation of PEG, relocation of medical resources, and improvement of PEG-related care.
